# Time Course of Endothelial Dysfunction Induced by Decompression Bubbles in Rats

**DOI:** 10.3389/fphys.2017.00181

**Published:** 2017-03-23

**Authors:** Kun Zhang, Mengmeng Wang, Haowen Wang, Yinuo Liu, Peter Buzzacott, Weigang Xu

**Affiliations:** ^1^Department of Diving and Hyperbaric Medicine, Faculty of Naval Medicine, Second Military Medical UniversityShanghai, China; ^2^School of Sports Science, Exercise and Health, University of Western AustraliaPerth, WA, Australia

**Keywords:** decompression sickness, bubbles, bubble amount, endothelial cells, decompression stress

## Abstract

Decompression stress can cause endothelial injury, leading to systematic inflammation and prothrombotic phenomena. Our previous work found that endothelial injury following decompression correlated positively with bubble formation. This study aimed to investigate the time course of endothelial injury and the relationship with bubble amounts. Rats were subjected to a simulated air dive to 7 ATA for 90 min with rapid decompression. Bubbles were detected ultrasonically at the root of pulmonary arteries following decompression. Surviving rats were randomly divided into six groups according to sampling time following decompression (2, 6, 12, 24, 48, and 72 h). Three parameters, serum levels of malondialdehyde (MDA), endothelin-1 (ET-1), and intercellular cell adhesion molecule-1 (ICAM-1) were identified from our previous study and measured. The level of MDA reached a peak level at 12 h post decompression, and then decreased gradually to control level before 72 h. For both ET-1 and ICAM-1, the greatest expression appeared at 24 h following surfacing, and the increases lasted for more than 72 h. These changes correlated positively with bubble counts at most detection time points. This study reveals the progress of endothelial dysfunction following decompression which provides guidance for timing the determination at least for the current model. The results further verify that bubbles are the causative agents of decompression induced endothelial damage and bubble amounts are an objective and suitable parameter to predict endothelial dysfunction. Most importantly, levels of endothelial biomarkers post dive may serve as sensitive parameters for assessing bubble load and decompression stress.

## Introduction

Inert gas supersaturation results in bubble formation in blood and tissue following rapid reduction in environmental pressure, causing decompression sickness (DCS) (Vann et al., 2011; Papadopoulou et al., 2013). Depending on their size, bubbles present in blood can circulate and even obstruct vessels (Papadopoulou et al., 2014). Residing at the vessel interface, vascular endothelial cells are vulnerable to intravascular bubbles (Jufri et al., 2015; Givens and Tzima, 2016). Damage to endothelial cells by decompression stress has been reported in a number of studies followed by synthesis of cytokines and cell adhesion stimulators, and finally systematic inflammation and prothrombotic phenomena(Bigley et al., 2008; Brubakk and Mollerlokken, 2009; Papadopoulou et al., 2014; Blatteau et al., 2015). Several drugs were confirmed to be preventive to DCS partially through their endothelial-protective properties(Møllerløkken et al., 2006; Ni et al., 2011; Zhang et al., 2014, 2016a; Blatteau et al., 2015). It is now widely accepted that endothelial injury plays a significant role in the progress of DCS (Levett and Millar, 2008; Brubakk and Mollerlokken, 2009; Klinger et al., 2011; Vann et al., 2011; Thom et al., 2015).

The pathophysiological etiology of endothelial injury following diving is not fully understood yet. Recently, we found that endothelial dysfunction correlated positively and linearly with bubble amounts in a rat DCS model, indicating a close relationship between bubble formation and endothelial injury (Zhang et al., 2016b). In the rat DCS model adopted in our study, bubble counts peaked at around 20~30 min post-dive and lasted for <2 h in most cases (Ni et al., 2013; Zhang et al., 2016b). However, evidence of damage to endothelial cells has been observed even for several days after decompression (Obad et al., 2007; Vince et al., 2009; Bilopavlovic et al., 2013), but no previous data describes the time course of endothelial injury biomarkers.

The aim of the present study was to investigate the progress of endothelial dysfunction following a simulated air dive with rapid decompression in rats, and to study the relationship with bubble formation, which was thought to be helpful in understanding diving-related endothelial dysfunction and providing guidance for timing the determination of endothelial injury.

## Materials and methods

### Animals

A total of 68 Sprague-Dawley male rats were used for the experiments. In order to eliminate the influence of age, all rats used in the experiment were 9–10 weeks old. The protocol was approved by the Animal Ethics Committee of Second Military Medical University and all procedures were carried out in accordance with the relevant guidelines. Rats were housed in a controlled environment with a 12/12-h light/dark cycle, constant temperature (23 ± 1°C) and relative humidity (54 ± 2%) during all the experiment, lived in pair and moved freely in rodent cages with food and water available *ad libitum*.

### Grouping and treatment

All the 68 rats were subjected to a simulated air dive to induce decompression stress and bubble formation. Four rats died shortly after the decompression were excluded from the study and the remaining 64 surviving rats were randomly divided into six groups according to sampling time (2, 6, 12, 24, 48, and 72 h, *n* = 4 for 2 h and *n* = 12 for the rest group) after decompression. The results of 8 rats in a previous study (Zhang et al., 2016b) were incorporated into the 2 h Group which received the same treatment. So, the number in each of the six groups is 12. The normal control results were also from the previous study, in which, 8 rats were sham exposed (normobaric air) in the same chamber for the same length of time. Bubbles flowing through the pulmonary artery were detected ultrasonically for 2 h after decompression. The rats in different groups were anesthetized and sacrificed at the respective time point after decompression for measurement of endothelial biomarkers.

### Simulated diving

The rats were compressed with air to 7 absolute atmospheres (ATA) in 5 min and maintained for 90 min before decompression in a transparent hyperbaric rodent chamber (Type RDC150-300-6, SMMU, Shanghai, China) using the same protocol as in our previous study (Zhang et al., 2016b). The rats were compressed at an increasing rate from 1 ATA/min to 1.5 ATA/min to minimize possible middle ear squeeze in the animals. To avoid carbon dioxide retention, carbon dioxide absorbent was used and the chamber was continuously ventilated during the exposure. Decompression was performed linearly to ambient pressure in 4 min, which has been proven in our previous study to induce detectable bubbles in the animals with a very low mortality (Zhang et al., 2016b).

### Bubble detection and grading

Immediately after decompression the rats were anesthetized with 3% pentobarbital sodium (1.5 ml/kg body weight, i.p.) (Sinopharm Chemical Regent Co., Shanghai, China) and were lain supine on a thermo-regulating pad (32°C). The anesthesia was lasted for 2 h during the whole bubble detection period and all the rats recovered shortly after the detection except the rats in 2 h group, which were sampled immediately after the detection. The fur on the chest was removed and bubble detection was performed in the cross-section at the root of the pulmonary artery using an ultrahigh frequency (18 MHz) detector connected to an ultrasonic scanner (Mylab30cv, Esaote, Italy). The delay between surfacing and ultrasonic detection of the pulmonary artery was 5 min or less. For every rat detection was repeated at 5, 10, 20, 30, 45, 60, 90, and 120 min after decompression, each lasting for 60 s (Zhang et al., 2016b). Bubbles were seen as moving bright spots in ultrasound images and the amounts were scored according to a grading system described elsewhere (Eftedal et al., 2007). The total bubble count for each rat indicates the detected number of bubbles flowing through pulmonary artery, which was presented by the area under the curve of bubble grade changes with time.

### Measurement of endothelial biomarkers

Rats were anesthetized and blood was drawn from the right ventricle under anesthesia and transfused into 2-ml Eppendorf tubes without anticoagulation. Then the samples were placed in room temperature for 2 h before centrifuging (1,000 × g, 20 min at 4°C). The supernatant was stored at −80°C until determination. Serum levels of endothelin-1 (ET-1) and intercellular cell adhesion molecule-1 (ICAM-1) were assayed by ELISA (Jiancheng Bioengineering Institute, Nanjing, China) (Liang et al., 2016; Yu et al., 2017). Levels of malondialdehyde (MDA) were detected by chemical colorimetry using commercial assay kits (Beyotime Institute of Biotechnology, Nantong, China) (Yang et al., 2015). All assays were performed in accordance with the respective manufacturer's instructions.

### Statistical analysis

Unless otherwise stated, all data are presented as mean±SD. Normal distribution was tested using a Kolmogorov-Smirnov test. One-way ANOVA followed by post hoc Student Newman–Keuls tests or Dunnett's tests were used for multiple comparisons between means. Pearson correlation was used for correlation analysis between endothelial parameters and bubble counts. The threshold for significance was accepted at *P* < 0.05.

## Results

### Bubble formation post decompression

**Figure 1:**
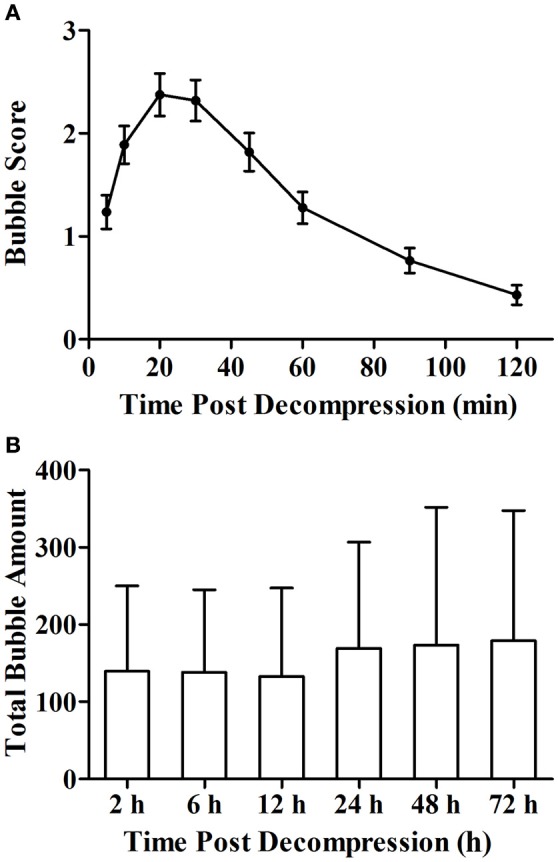
**Bubble formation following decompression (A)** and total bubble amounts of rats in each group **(B)**. Rats were compressed with air to 7 ATA and maintained for 90 min before linear decompression to atmospheric pressure in 4 min. After decompression, bubbles were detected and scored at the 8 time points with 2 h. Error bars are standard error of the mean (SEM). In **(B)**, total bubble count was calculated as the area under the curve similar to that shown in **(A)** for each rat, and no statistical difference was detected between the groups. *n* = 72 for Figure **(A)**, *n* = 12 for each group in **(B)**.

The simulated air dive induced bubble formation in all rats. Bubble counts increased gradually following decompression and reaching a maximum at around 20 min (Figure 1A). Bubbles disappeared within 2 h in most rats. The total bubble count for each rat varied widely between the animals at each time point (Figure 1B). There was no difference in the total bubble score between groups (*P* > 0.05).

### Time course of endothelial biomarkers following decompression

**Figure 2:**
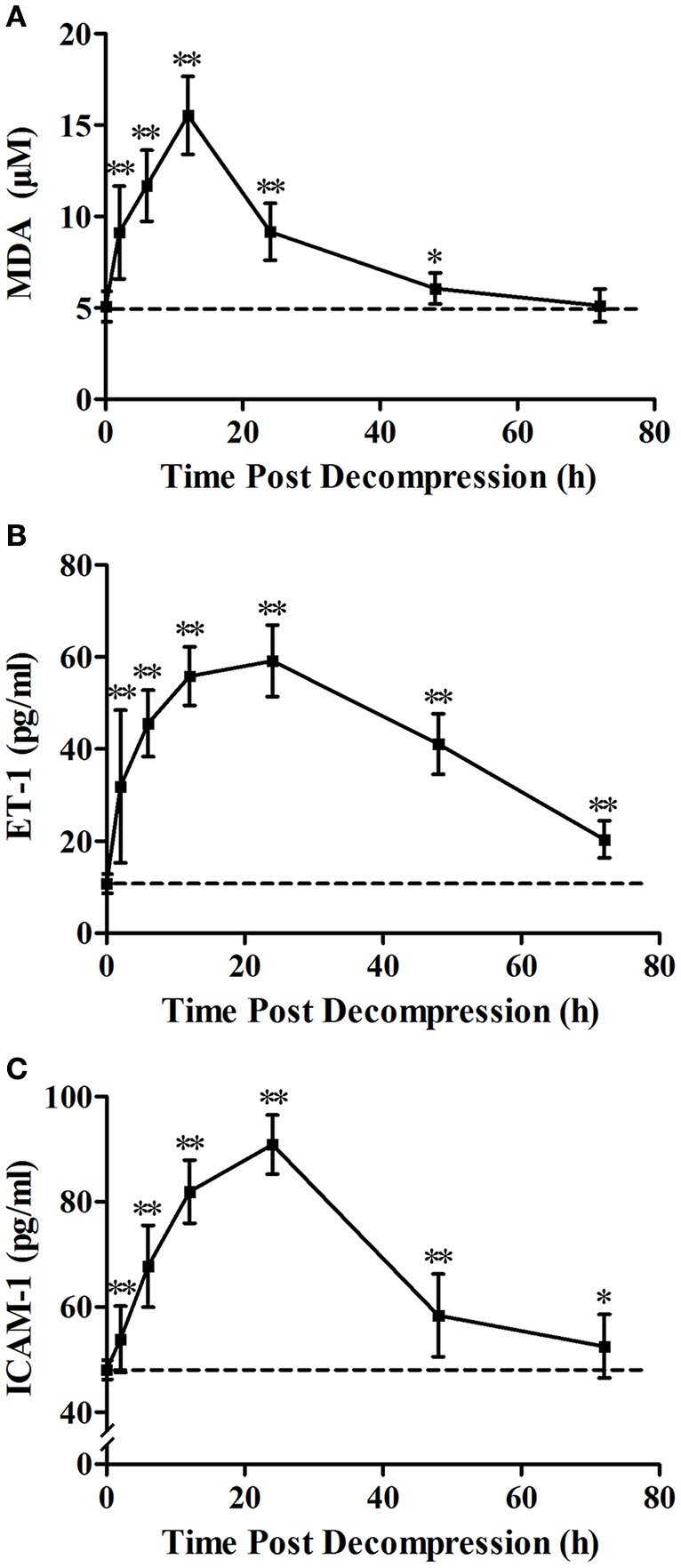
**Time course of decompression induced changes in MDA (A)**, ET-1 **(B)** and ICAM-1 **(C)**. Rats were anesthetized and sampled at 2, 6, 12, 24, 48, and 72 h after decompression from an air dive (7 ATA-90 min) in 4 min. 0 h denotes Normal control group and the broken line indicates the mean Normal control group value. *n* = 8 for Normal control group, *n* = 12 for each DCS modeling group. ^**^P < 0.01, ^*^P < 0.05 vs. Normal control group.

Levels of MDA, ET-1, and ICAM-1 gradually increased after decompression and peaked at 12, 24, and 24 h, respectively (Figure 2). The level of MDA decreased to the Normal control level before72 h (*P* = 0.889). Compared with normal values, levels of ET-1 and ICAM-1 were significantly increased over the 72 h observation period (*P* < 0.05) with an obvious recovery trend after 24 h.

### Relationship between endothelial biomarkers and bubble amount

**Figure 3:**
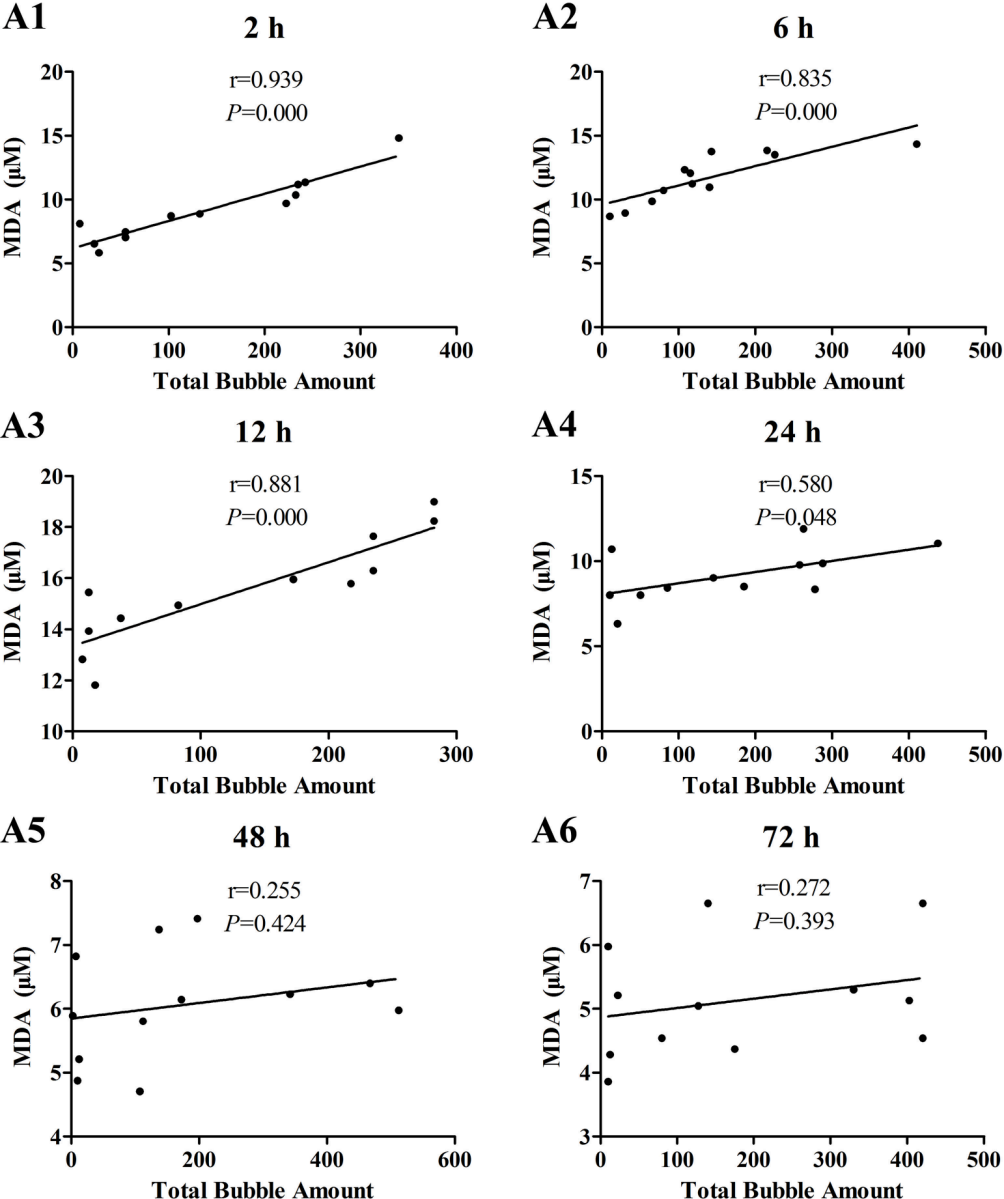
**Correlation between MDA levels and total bubble amounts**. MDA was detected at 2 **(A1)**, 6 **(A2)**, 12 **(A3)**, 24 **(A4)**, 48 **(A5)**, and 72 h **(A6)** after decompression in 4 min from a simulated air dive (7 ATA-90 min). Total bubble count which indicates the detected number of bubbles flowing through the pulmonary artery was calculated as the area under the curve shown in Figure 1A. *n* = 12.

**Figure 4:**
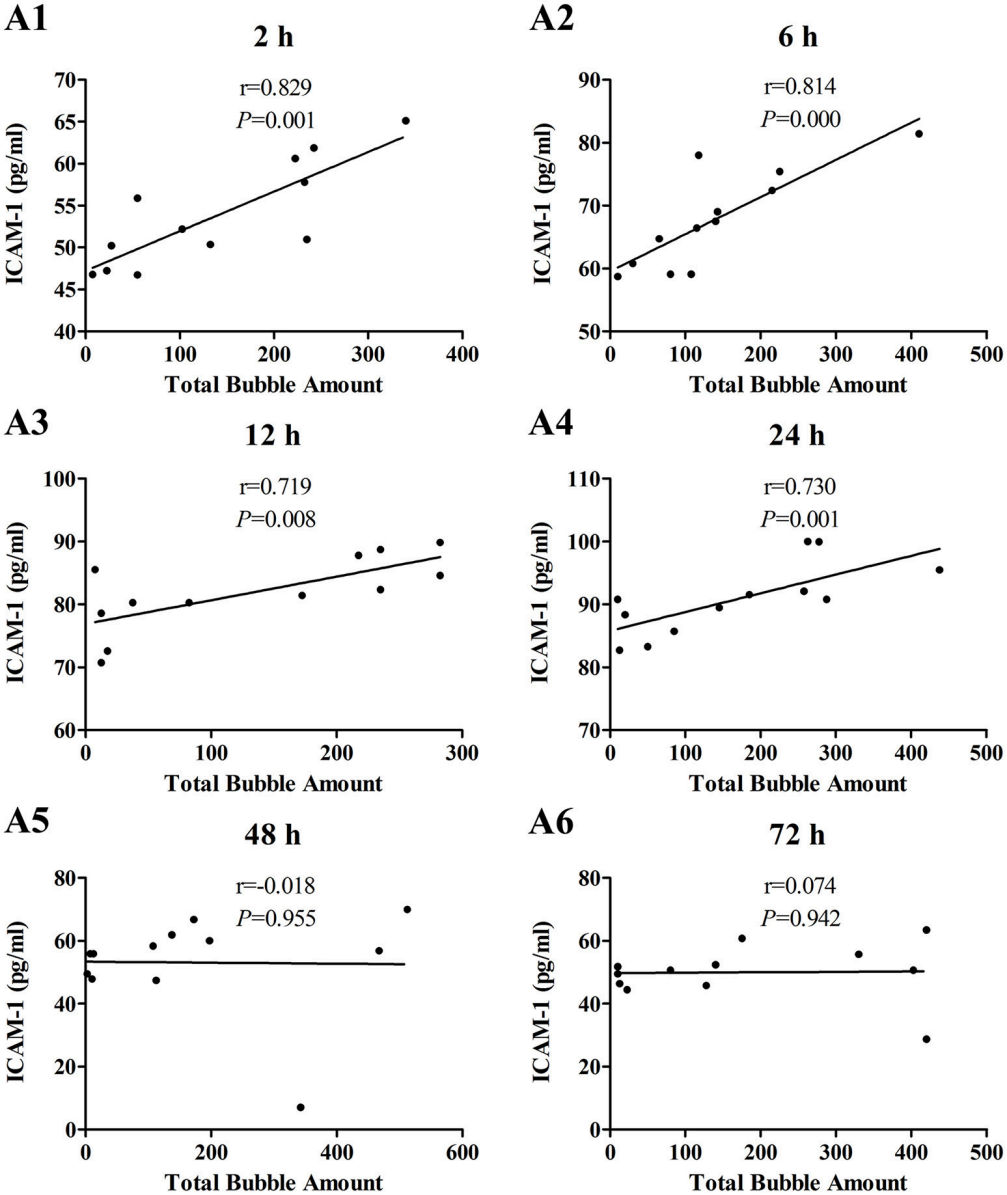
**Correlation between ICAM-1 levels and total bubble amounts**. ICAM-1 was detected at 2 **(A1)**, 6 **(A2)**, 12 **(A3)**, 24 **(A4)**, 48 **(A5)**, and 72 h **(A6)** after decompression in 4 min from a simulated air dive (7 ATA-90 min). Total bubble count which indicates the detected number of bubbles flowing through the pulmonary artery was calculated as the area under the curve shown in Figure 1A. *n* = 12.

**Figure 5:**
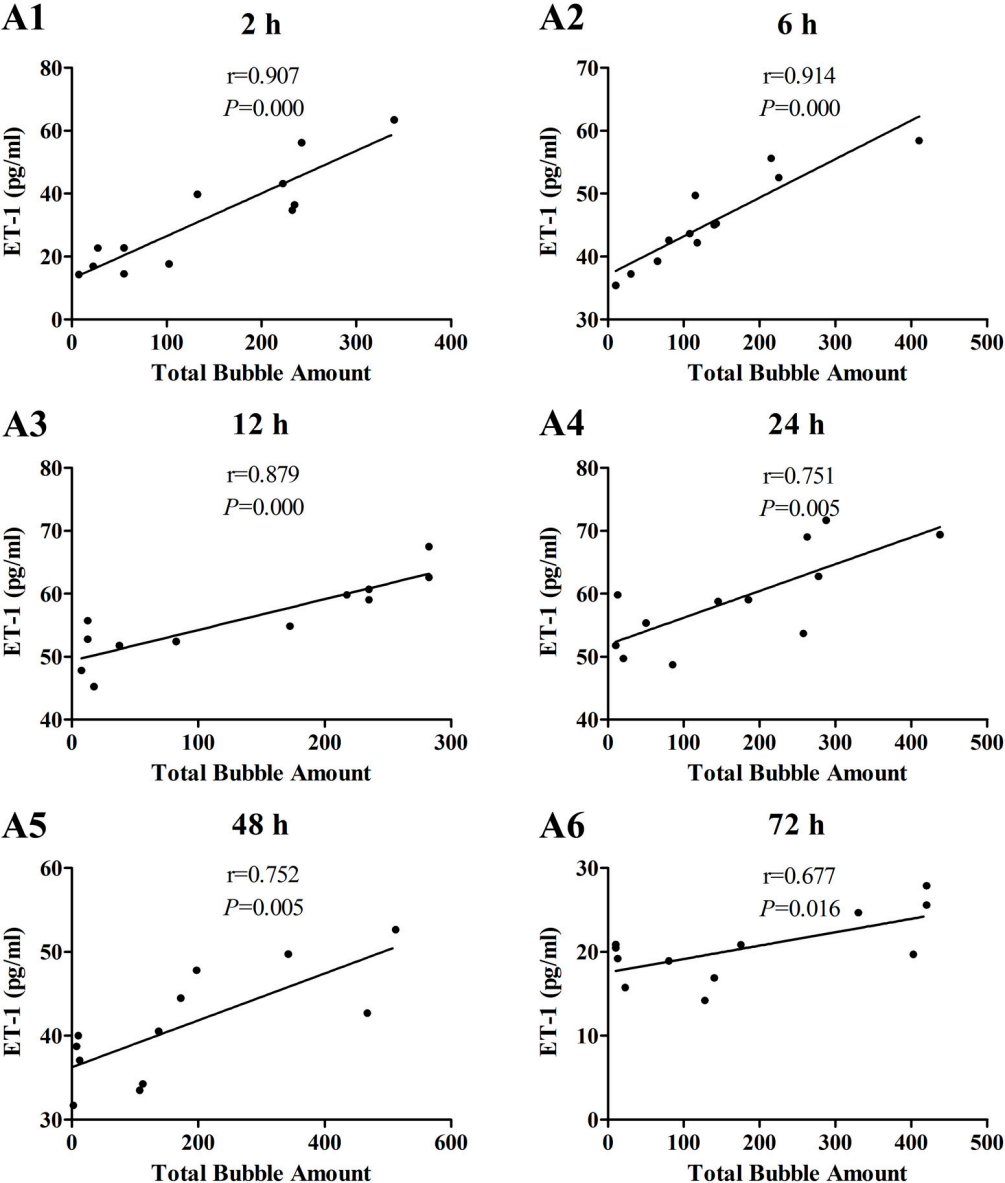
**Correlation between ET-1 levels and total bubble amounts**. ET-1 was detected at 2 **(A1)**, 6 **(A2)**, 12 **(A3)**, 24 **(A4)**, 48 **(A5)**, and 72 h **(A6)** after decompression in 4 min from a simulated dive (7 ATA-90 min). Total bubble count which indicates the detected number of bubbles flowing through the pulmonary artery was calculated as the area under the curve shown in Figure 1A. *n* = 12.

The increases of MDA and ICAM-1 correlated positively with bubble counts within 24 h (*P* < 0.05, Figures 3, 4). For ET-1, the increase significantly correlated with bubble counts over the entire observation period (*P* < 0.05, Figure 5).Reference levels of the biomarkers at each bubble grade are listed in Table 1. The results in the 2 h time point group include those obtained from 24 rats in the above mentioned study (Zhang et al., 2016b).

**Table 1:** **The reference levels of endothelial biomarkers at different bubble grades in a rat DCS model**.

**Bubble grade**	**Serum MDA (μM) (95% CI)**					
	**2 h**	**6 h**	**12 h**	**24 h**	**48 h**	**72 h**
0	4.37-5.77	4.37-5.77	4.37-5.77	4.37-5.77	4.37-5.77	4.37-5.77
~1	5.63-7.47	9.21-11.87	12.47-15.33	6.33-10.25	4.73-6.37	3.74-5.81
~2	8.45-10.27	10.83-15.20	15.08-17.76	5.54-11.99	5.22-8.64	2.44-8.27
~3	10.60-13.78	//	13.78-23.75	7.65-12.29	/	/
~4	/	/	//	/	/	2.73-8.15
~5	//	//	//	//	/	//
	**Serum ET-1 (pg/ml) (95% CI)**					
0	9.07-12.53	9.07-12.53	9.07-12.53	9.07-12.53	9.07-12.53	9.07-12.53
~1	13.72-23.01	37.06-45.81	47.00-54.93	47.43-58.72	32.50-39.29	16.57-21.37
~2	33.51-39.70	41.15-58.05	54.46-62.74	57.30-60.55	35.22-53.33	9.03-25.63
~3	52.07-61.52	//	34.22-95.89	51.56-77.01	/	/
~4	/	/	//	/	/	13.92-34.87
~5	//	//	//	//	/	//
	**Serum ICAM-1 (pg/ml) (95% CI)**					
0	46.56-49.55	46.56-49.55	46.56-49.55	46.56-49.55	46.56-49.55	46.56-49.55
~1	47.46-52.22	57.41-70.26	72.26-83.74	81.94-90.40	47.50-57.49	44.75-52.35
~2	50.89-57.00	65.48-76.70	79.14-90.98	77.40-103.61	54.25-71.56	34.28-71.65
~3	59.41-65.23	//	53.86-120.58	87.82-103.59	/	/
~4	/	/	//	/	/	41.57-73.61
~5	//	//	//	//	/	//

*Serum levels of MDA, ET-1 and ICAM-1 were determined in rats at 2, 6, 12, 24, 48, and 72 h after decompression in 4 min from a simulated air dive (7 ATA-90 min). Bubbles were repeatedly detected within 2 h following decompression, and the mean grade was calculated for the 2 h period. The results of another 16 rats (not the same as those referred in Method section) from a previous study were included in the 2 h time point group. n = 28 for 2 h and n = 12 for the rest sampling time point groups. n = 8 for normal control group (bubble grade 0). /: only one value which could not be used to calculate 95% confidence interval (CI); //: no data*.

## Discussion

Vascular endothelial cells are well-described targets for decompression stress and endothelial injury plays an important role in the process of DCS (Lambrechts et al., 2013; Mazur et al., 2014, 2016; Fok et al., 2015; Wang et al., 2015), though the exact mechanism remains unclear. Whether bubbles are the cause or not, endothelial dysfunction is detectable and obvious following most diving exposures (Madden and Laden, 2009; Chrismas et al., 2010; Klinger et al., 2011; Papadopoulou et al., 2014). To further study the time course of endothelial dysfunction will help better understand the pathophysiology of decompression injuries and provide additional evidence in establishing the etiology.

Three decompression rates were adopted in our previous study to induce varying amounts of bubbles, and also to study the effects of decompression *per se* on endothelial cells (Zhang et al., 2016b). Among the three rates, 4 min decompression from 60 msw (15 msw/min) to atmospheric pressure yielded low mortality (5% in the current study), and yet still induced a wide range of bubble formation. Limited by the blood volume of rats and micro-sampling analysis techniques, multi-sampling over the 72 h period could not be achieved in a same animal, thus involving a greater number of animals and adding variation between groups. Satisfactorily, the total bubble amounts were similar between the six groups for the different sampling time-points during the 72 h period, which meets the basic statistical criteria for comparing biomarkers between groups.

The three biomarkers of endothelial dysfunction and decompression stress, MDA, ET-1 and ICAM-1, were selected from the nine parameters determined in our previous study (Zhang et al., 2016b); all three biomarkers changed sensitively and correlated better with bubble amounts at 2 h following rapid decompression. Among them, ICAM-1 is a sensitive biomarker with the capacity to reflect endothelial damage directly; ET-1 is a kind of vasoactive substance secreted by endothelial cells which could also reflect endothelial damage; MDA serves as a sensitive biomarker of oxidative stress which was involved in the pathogenesis of endothelial injury after decompression (Zhang et al., 2016b). This was confirmed again in the present study. Rapid decompression induced significant increases in all these parameters, indicating obvious endothelial damage. For MDA, it reached a peak level (3.1 fold of mean normal control group values) at 12 h post decompression, and decreased gradually to normal control group levelsbefore72 h. For both ET-1 and ICAM-1, the greatest expression appeared at 24 h following surfacing (5.5 and 1.9 times normal control group values, respectively), and these increases lasted for more than 72 h. Although the changes after 72 h were not determined, it could be imagined that levels of ET-1 and ICAM-1 would recover to normal before 96 h post dive from the trend showed in Figure 2.

The time course provided necessary information for timing the determination of DCS endothelial injury. From the curves presented in Figure 2, it can be seen that the duration of half-elevation levels for MDA, ET-1, and ICAM-1 were around 2–24, 2–60, and 6–40 h, respectively. During these periods, these biomarkers provide good assessment of bubble loads and decompression stress. From the results shown in Figures 2–5, ET-1 had the best capability in reflecting bubble load and decompression stress.

The increase in all biomarkers correlated well with bubble counts at most of the detection time points (Figures 3–5), which is in accordance with previous studies (Nossum et al., 1999, 2002; Zhang et al., 2016b). More bubble formation caused more serious injury to endothelial cells in a dose-response relationship. As shown in Table 1, from the increased value of the biomarkers, bubble grades could be estimated. In the present study however, limited by the number of animals used for each group, the values at each time point post-dive need further repetition to estimate confidence intervals. The results from later studies should be added to this table, and similar works are warranted for big animal DCS models and eventually, for divers.

One other finding that can be seen in Figures 3–5 is that the correlations between biomarkers and bubbles for all the three parameters have a decreasing trend with time even before the peak responses, showed by the gradual decreasing in correlation coefficient (*r*) values. Therefore, earlier determination of the biomarkers started at 2–6 h post decompression likely offers better estimation of decompression stress and bubble formation.

Although the decompression induced intravascular bubbles existed for <2 h, the damage to endothelial cells was on-going and progressed to peak during 12–24 h after the disappearance of bubbles. Many chemical drugs, including simvastatin, hydrogen, and NO donors, have been suggested to have prophylactic effects against DCS due to their endothelial-protective properties (Møllerløkken et al., 2006; Ni et al., 2011; Zhang et al., 2014, 2016a; Wang et al., 2015). Based on the current findings, it is reasonable to hypothesize that endothelial protective agents can be not only administered prior to a risky dive, but also can be used as therapeutic approaches in DCS treatment immediately after dives.

Intravascular bubbles can damage endothelial cells directly by mechanical contact or indirectly via initiating biochemical cascades, e.g., coagulation activation, inflammatory responses, and oxidative stress, or other effects of bubble-endothelial cell interactions (Nossum et al., 1999, 2002; Suzuki and Eckmann, 2003; Suzuki et al., 2004; Eftedal et al., 2007; Sobolewski et al., 2012; Papadopoulou et al., 2014; Blatteau et al., 2015; Wang et al., 2015). Injured endothelial cells may even release other kinds of substances and initiate further endothelial injury remotely (Chrismas et al., 2010; Thom et al., 2012). The present results confirm our previous finding that endothelial dysfunction correlates well with bubble formation (Zhang et al., 2016b). No matter whether the injury was directly or indirectly from bubbles, bubbles are the most likely initial causative agents of endothelial dysfunction following diving decompression.

In conclusion, the present study highlighted the time course of endothelial injury together with its relationship with bubble amounts. These results provide guidance for timing endothelial dysfunction following diving, at least for the current animal model. The results also reconfirm that bubble amounts are an objective and suitable parameter to predict endothelial dysfunction; and most importantly, the levels of endothelial biomarkers post diving might serve as simple yet sensitive parameters in the assessment of bubble load and decompression stress. ET-1, MDA, and ICAM-1 were further proven to be sensitive biomarkers with the capacity to indicate endothelial dysfunction and decompression stress. Further studies on divers are needed which may improve the assessment of decompression stress and explore the etiology, prevention and treatment of decompression sickness.

## Author contributions

WX and KZ designed and KZ, MW, HW, and YL conducted the experiments. All authors listed contributed to data analyses and interpretation of the results. WX, KZ, and PB wrote the manuscript. KZ and WX prepared all the figures and the table. KZ and MW contributed equally to this work. All authors reviewed the manuscript and agreed to be accountable for the content of the work.

## Funding

This work was supported by the National Natural Science Foundation of China No. 81571846.

### Conflict of interest statement

The authors declare that the research was conducted in the absence of any commercial or financial relationships that could be construed as a potential conflict of interest. In addition to the listed affiliations, Peter Buzzacott is also employed by Divers Alert Network as Director of Injury Monitoring and Prevention but that employment bore no relation to this study.
